# The Potential Effect of Changing Patient Position on Snoring: A Systematic Review

**DOI:** 10.3390/jpm14070715

**Published:** 2024-07-02

**Authors:** Antonio Moffa, Lucrezia Giorgi, Domiziana Nardelli, Francesco Iafrati, Giannicola Iannella, Giuseppe Magliulo, Peter Baptista, Claudio Vicini, Manuele Casale

**Affiliations:** 1 School of Medicine, Università Campus Bio-Medico di Roma, 00128 Rome, Italy; 2 Integrated Therapies in Otolaryngology, Fondazione Policlinico Universitario Campus Bio-Medico, 00128 Rome, Italy; 3 Unit of Measurements and Biomedical Instrumentation, Department of Engineering, Università Campus Bio-Medico di Roma, 00128 Rome, Italy; 4 Department of Organi di Senso, University Sapienza, 00185 Rome, Italy; 5 ENT Department, Al Zahra Private Hospital Dubai, Dubai 23614, United Arab Emirates; 6 ENT and Audiology Department, University of Ferrara, 44121 Ferrara, Italy

**Keywords:** snoring, positional therapy, OSA, HOBE, treatment

## Abstract

Approximately 45% of adults snore occasionally, and 25% snore regularly, with a higher prevalence in men and an increase among postmenopausal women due to hormonal changes. Snoring is a health concern linked to vascular disease and decreased quality of life for both snorers and their bed partners. Effective snoring treatment, which aims to reduce or eliminate the sound, is challenging and depends on factors like age, comorbidities, disease severity, and anatomical features. This review aims to provide a systematic overview of the current literature on the effects of positional therapy (PT) on snoring. Various devices facilitate PT, including anti-snoring pillows and vibration alarms. PT devices maintain head and neck alignment to keep airways open, while head of bed elevation (HOBE) solutions reduce upper airway collapses by elevating the head and trunk. Studies show that PT and HOBE reduce snoring by increasing airway cross-sectional area and decreasing closing pressure. Despite their benefits, these non-surgical treatments have limitations, such as discomfort in certain sleeping positions and intolerance to prolonged head elevation. While reducing snoring intensity is critical for health reasons, further comparative studies between the different devices are needed to enhance snoring management.

## 1. Introduction

Snoring is a widely recognized common symptom among patients with obstructive sleep apnea (OSA). However, it is not universally true that individuals who snore necessarily have OSA. Snoring stands as the primary nocturnal OSA symptom [1]. Indeed, an estimated 45% of adults snore only occasionally, while 25% snore regularly [2]. There is a notable gender discrepancy; 40% of snorers are men, while 24% are women [3,4,5]. In the female population, the incidence of snoring increases in the postmenopausal period, probably due to hormonal changes [6,7]. Snoring is not only a “cosmetic” problem, but it can also pose a health risk and impact quality of life; habitual snorers are more susceptible to vascular disease [8].

Snoring every night can lead to fragmented sleep, reducing overall sleep quality and causing daytime fatigue, irritability, and decreased cognitive function. Moreover, snoring has been associated with multiple subclinical markers of cardiovascular pathology, including elevated blood pressure, increased carotid-intima-media thickness, stenosis, and atherosclerosis. These effects could partly reflect mechanical stress imposed by snoring vibrations on the UA in combination with a range of shared risk factors for OSA and cardiovascular disease, such as obesity and a sedentary lifestyle [9,10].

Snoring without sleep apnea could have negative consequences on the individual’s bed partners, family members, and general quality of life [7]. Snoring typically arises during inspiration because of increased upper airway (UA) tract resistance during sleep. This resistance rises as the UA narrows, leading to vibration and the characteristic snoring sound [8]. The sound of snoring commonly originates from abnormalities in the soft palate or uvula. When airflow passes through the airway, a long or floppy soft palate can vibrate, generating the sound of snoring.

Several therapy options are available for snoring, and choosing the proper treatment can be difficult. Snoring treatment remains a challenge for ENT. Numerous variables need to be taken into account, including the patient’s age, comorbidities, disease severity, palate phenotypes, the presence of OSA, and upper airway anatomical features [9,10]

The main goal of snoring treatment is to reduce the sound intensity or, if possible, eliminate it completely. In cases of concurrent OSA, an effective treatment to eliminate apnea should be chosen. These solutions include both surgical and non-surgical options. There are several surgical options for snoring both under local anesthesia, such as injection sclerotherapy, laser therapy, radiofrequency ablation, palatal implants, and under general anesthesia with new remodeling intrapharyngeal surgery. Recently, many barbed pharyngoplasties have been shown to reduce OSA and improve snoring [11] and it seems to be effective also in simple snorers [3].

However, very frequently, especially in the absence of apnea, many patients do not want to undergo surgical procedures and wish to undertake a non-surgical option. As the first approach, in order to reduce snoring, physicians could suggest, if applicable, weight loss and other lifestyle modifications, such as the avoidance of alcohol and sedatives. Among non-surgical treatments, there is changing position. In particular, positional therapy (PT) appears to be an effective method of treating snoring in selected patients. The severity of snoring is more pronounced in the supine sleeping position. Therefore, PT devices create a physical barrier to prevent a supine position during the night through an electrical vibration when the supine position is detected. Several PT devices are currently available. PT devices have been evaluated in non-randomized studies to assess their effect on snoring and described in RCTs in positional OSA. In order to improve snoring and OSA, avoiding the supine position is not the only solution among different change positions during the night [12]. Recently, the head-of-bed elevation position (HOBE), also known as Fowler’s position, has been proposed. This standardized position involves the patient sleeping in a semi-recumbent position with an incline ranging between 30° and 60° [12,13]. This position is associated with better chest expansion and improved breathing by facilitating blood oxygenation, making it a valid PT option that does not disrupt sleep architecture. Before finding its application in snoring and OSA, it was deemed a valid option in several other pathologies, including gastroesophageal reflux [14] or in improving respiratory patterns in patients under mechanical ventilation [15,16]. The actual impact of PT and HOBE, in comparison to the supine position, on reducing OSA events, particularly snoring, collapsibility of the mucous membranes, and UA area, has not been thoroughly studied in a large number of physiological and clinical studies.

This review provides a systematic overview of the current literature on the effects of changing position on snoring, regardless of whether the patients are OSA patients or simply snorers.

## 2. Materials and Methods

### 2.1. General Study Design

The study was designed following the recommendations of the Centre for Review and Dissemination’s Guidance for Under-taking Review in Health Care and is reported in adherence of the Preferred Reporting Items for Systematic Review and Meta-Analyses (PRISMA) statement [17]. The systematic review is registered in PROSPERO (Registration ID: CRD42024559488).

### 2.2. Data Source and Study Searching

An electronic search was performed on the PubMed/MEDLINE, Google Scholar, and SCOPUS databases. An example of a search strategy is the one used for PubMed/MEDLINE: “Positional therapy” and “Snoring”; “Position-dependent snoring” and “Therapy”; “Pillow” and “Snoring”; “Simple-snoring” and “positional therapy”; “Sleep positioning pillow” and “Snoring”; “Anti-snoring pillow”; “Head of bed elevation” and “Snoring”; “Inclined position” and “Snoring”. All the searches were adjusted to fit the specific requirements for each database, with a cross-reference search to minimize the risk of missing relevant data. The last search was performed in May 2024.

### 2.3. Inclusion/Exclusion Criteria

According to the PICOS acronym [18], we included the studies with the following characteristics: patients (P), adult patients affected by snoring with and without OSA; intervention (I), positional therapy and head-of-bed-elevation; comparison (C), pretreatment and post-treatment; outcome (O), snoring index, snoring intensity, self-reported questionnaire (e.g., snoring VAS, Epworth Sleepiness Scale (ESS), Pittsburgh Sleep Quality Index (PSQI), Function Outcomes of Sleep Questionnaire (FOSQ)); and study design (S), both prospective and retrospective cohort studies. The exclusion criteria were: (1) studies not written in English; (2) case reports, reviews, conference abstracts, and letters; (3) studies with unclear and/or incomplete data; (4) studies evaluating only the effects of positional therapy for the treatment of OSA and not of snoring; (5) pediatric studies; and (6) studies published before 2009 to discuss the solution of the last 15 years.

### 2.4. Data Extraction and Data Analysis

The articles were first screened based on their titles and abstracts. Next, the full-text versions of each publication were evaluated, and those deemed irrelevant to the review’s topic were excluded. Data extraction from the included studies was conducted systematically using a structured form. A qualitative synthesis was performed on the selected studies to analyze the effects of positional therapy on snoring.

### 2.5. Statistical Analysis and Summary of Findings

Due to the heterogeneous reporting styles and insufficient data in the included studies, conducting a statistical analysis or providing a quantitative summary of the findings was not feasible. Therefore, the effects on individual outcomes and the overall quality assessments were described narratively. The authors of the included studies were not contacted for additional information.

## 3. Results

**Figure 1:**
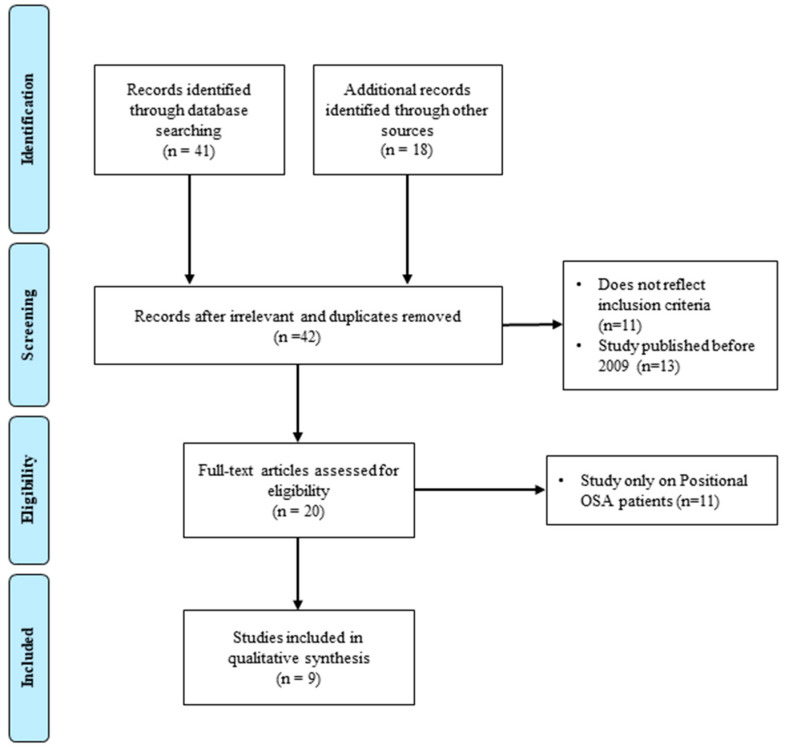
Flowchart outlining the paper selection process of the systematic review (based on PRISMA guidelines).

The search criteria initially returned 59 articles, from which 17 papers were excluded due to irrelevance or duplication. Further screening led to the exclusion of an additional 33 papers, leaving nine articles that met the inclusion criteria. The selection process is illustrated in Figure 1 (PRISMA flow diagram). The included studies involved a total of 235 patients, aged between 21 and 72 years. The characteristics of these studies are detailed in Table 1, with further descriptions available in Table 2. Among the included patients, only 50 were identified as simple snorers, while the remaining patients suffered from mild to severe OSA.

### 3.1. Supine Alarm Device

Benoist et al. [19] enrolled 30 snorers with a sleep position trainer (SPT) at home for six weeks. The SPT was a lightweight and small device (72 × 35 × 10 mm, 25 g), worn around the chest with a neoprene strap, able to give a subtle vibration when in the supine position. After six weeks of therapy, the authors showed a significant improvement in the Snore Outcome Survey (from 35.0 ± 13.5 to 55.3 ± 18.6; *p* < 0.001), in the Spouse/Bed Partner Survey (from 24.7 ± 16.0 to 54.5 ± 25.2; *p* < 0.001), and in snoring assessed with a numeric visual analog scale (VAS) by the bed partner (from a median of 8.0 with an interquartile range (IQR) of [7.0–8.5] to 7.0 [3.8–8.0]).

Bignold et al. [20] evaluated the efficacy of a supine alarm device (Buzz-POD^®^, Gorman ProMed Pty Ltd., Victoria, Australia) on 15 POSA patients who were also snorers (AHI ≥ 15 per hour, supine AHI twice or greater than the non-supine AHI; ≥20 min of sleep in supine and non-supine postures and non-supine AHI < 15). Patients were allocated to receive either one week of active PT or inactive PT in a random order, interposed by one week of washout before starting the other treatment. While the device demonstrated its efficacy in decreasing AHI by 45%, there were no differences in snore time nor bed partner snoring scale assessments. Moreover, bed partners’ sleep disturbance from the vibration alarm increased slightly but significantly (*p* = 0.01), corresponding to “a little” disturbance.

Choi et al. [21] studied 28 subjects diagnosed as position-dependent snorers with or without mild OSA. They used a vest-type device (Bio Sleep Medical, Seoul, Republic of Korea) with a connected controller and two 5 mm air tubes. Sleep time in the supine and non-supine positions significantly differed postexamination compared to the baseline (121.3 ± 70.4 min vs. 183.8 ± 80.2 min *p* < 0.0001, respectively). The total snoring rate (%) at baseline examination of 36.7 ± 20.6 decreased to 15.7 ± 16.2 (*p* < 0.0001).

### 3.2. Pillows

Cazan et al. [22] enrolled 22 adult snorers without OSA and without daytime sleepiness with BMI ≤ 30 and a bed partner. The Sissel^®^ SILENCIUM^®^ (Sissel Novacare, Bad Dürkheim, Germany) polyurethane foam pillow, featuring two integrated microphones, a sensor foil for head position detection, and two × six air chambers that could be individually targeted by a pneumatic system, was put to the test for four weeks. The snoring index at PSG (from 269 ± 249.7 to 162.5 ± 169.8) and the bed partners’ snoring VAS (7.2 versus 4.0) both significantly improved, according to the authors. Chung et al. [23] tested a smart anti-snoring pillow (SAP) containing a shift control assembly base and mobile foam. The mobile foam can shift horizontally back and forth automatically after detecting a person’s snoring sound, thereby changing their head and/or neck position. They included thirty patients (23 mild-to-moderate OSA and seven severe OSA patients) and found that the pre and post-therapy snore index (events/hour) reduced significantly from 501.5 ± 235.1 to 360.9 ± 21.1 (*p* = 0.003), and AHI (events/hour) improved from 21.8 ± 15.7 to 16.5 ± 17.8 (*p* = 0.001). The SAP significantly decreased the ODI, snoring number, snore index, and total and supine AHI in the mild-to-moderate OSA group but had no significant effect in the severe OSA group. Importantly, the action of the SAP is limited by the position of the patient. Indeed, the authors stated that when the person’s trunk was in the prone or lateral position, the SAP motion would not change the head and neck position.

Chen et al. [24] performed a study on 25 patients with snoring and positional OSA. On the first night (N0), the patients used regular pillows at home; on the second and third nights (N1 and N2), they used the head-positioning pillow (HPP), which was the Power Sleep^®^ anti-snore pillow (Green-Sweet Mattress Corp., New Taipei City, Taiwan). The HPP encourages the head to turn into the lateral sleep position because its median portion is narrower than the lateral sleep part. Because of this, subjects typically sleep in the lateral position for longer periods of time than in the supine position. When comparing the N0 value of 5.0 to the N2 value of 4.0 (*p* < 0.001; power = 98%), the median snoring severity dropped by 33.3%. Furthermore, from a N0 value of 218.0 events/h to a N2 value of 115.0 events/h (*p* = 0.001; power = 90%), the median snoring index dropped by 34.4%. There was no dose-dependent difference in the amount that the snoring severity and snoring index were reduced, according to BMI.

In the study conducted by Newel et al., [25] 28 patients with Positional OSA were enrolled in a prospective cohort study. The natural memory foam used in the Posiform^®^ sleep positioning pillow (Oscimed S.A. (Inc.)™, La Chaux-de-Fonds, Switzerland) is hidden beneath a detachable cotton cover and a second layer of velvet bamboo that reduces sweating. It has two inclined concave and flattened surfaces, a neck support, a frontal support, and a recess for the mouth, nose, and shoulder in addition to the central ridge. Patients performed a PSG during the first night and another one at one month and six months. Statistically significant changes were seen in total supine sleep time (%) (from 52 ± 21.2 to 20.4 ± 19.6, *p* = 0.001) and in AHI (from 12.1 ± 3.8 to 6 ± 3.5, *p* = 0.000). On the other hand, snoring time (%) improved, but the change did not reach statistical significance (26.3 ± 18.6 to 20.4 ± 22.6).

### 3.3. HOBE

Iannella et al. demonstrated the efficacy of HOBE with a 30° elevation of the head and trunk in improving UA obstruction in a population of 45 OSA patients, with a significant effect on snoring percentage, which decreased from 17.3 ± 11.5 to 12.5 ± 12.6 (*p* = 0.05) [12].

While the first study was performed in controlled settings, Lee and colleagues ran a pilot randomized controlled trial in daily life settings, this time using a mild HOBE (7.5° elevation) [26]. The inclination was delivered using a commercially available electric bed (Pharaoh Motion Care, BODYFRIEND Co., Ltd., Seoul, Republic of Korea). Both AHI and RDI were significantly decreased in OSA patients (*p* = 0.031 and *p* = 0.024, respectively). Snoring, however, was markedly improved but not in a range of statistical significance (*p* = 0.065).

Danoff-Burg et al. [27] studied 25 snorers for eight weeks. The patients used their own mattresses for four weeks in a flat position. After that, the Dr. Oz Good Life Adjustable Base Pro was placed in the place of their old bed base, enabling the patients to sleep on their original mattress for four weeks in an inclined position of twelve degrees. The applications “Do I Snore or Grind” and “Sleep Score Labs” were used to quantify subjective snoring. When sleeping in an inclined position as opposed to a flat position, there was a 7% relative reduction in snoring duration (*p* = 0.001), according to an objective measurement of snoring from night to night. Participants felt they snored less frequently (*p* = 0.01) and were awakened by their snoring less frequently (*p* < 0.001), according to self-report data. Participants with a bed partner (*n* = 10) reported that their partner woke them less often to stop snoring (*p* < 0.01).

## 4. Discussion

This article focused solely on studies investigating the effect of changing patient position on snoring, specifically in reducing nocturnal snoring, including snorers and OSA patients. Studies evaluating the effects of PT or HOBE in reducing only the OSA burden were excluded.

According to the included studies, PT and HOBE can be used to reduce nocturnal snoring, as shown by the snoring index, snoring time, the percentage of total sleep time spent in the supine position, and bed partners’ sleep disturbance. Both PT and HOBE represent cost-effective solutions that can be employed from the very beginning, being considerably affordable compared to CPAP equipment.

PT can be delivered with the help of several devices. Among them are anti-snoring pillows, including the Sissel^®^ SILENCIUM^®^ pillow (Sissel Novacare, Bad Dürkheim, Germany) [22], the Power Sleep^®^ anti-snore pillow (Green-Sweet Mattress Corp., New Taipei City, Taiwan) [24], and the Posiform^®^ pillow (Oscimed S.A. (Inc.)™, La Chaux-de-Fonds, Switzerland) [25]. All of them promote head and neck maintenance in the sniffing position [28] (head extension and neck flexion over the trunk) to align the upper respiratory tract and correctly open the upper airways. While the SAP and the SILENCIUM^®^ pillow (Sissel Novacare, Bad Dürkheim, Germany) dynamically adjust the head position by imperceptibly inflating air into air chambers, the Power Sleep^®^ pillow and the Posiform^®^ come with a fixed shape. Interestingly, the Power Sleep^®^ height can be selected on the widest retroglossal space achieved during flexible nasopharyngoscope examination.

Iannella et al. [12] enquired about the efficacy of a further category of device ensuring a 30° head and trunk inclination, known as HOBE positioning. They demonstrated a decrease in the incidence of total oropharynx lateral wall obstruction from 60% to 33.3% of cases (*p* = 0.01) and a decrease in the presence of total velum collapse from 82.3% of cases in the 0° supine position to 57.7% of cases (*p* = 0.02) using the HOBE position during DISE evaluation. The collapse of the tongue base and the epiglottis did not, however, improve statistically significantly when the patients were transferred from the supine position to the 30° HOBE position. In fact, UA collapses can be decreased by having the head and trunk positioned in the HOBE manner, showing a positive effect on snoring with a reduction of the percentage of snoring from 17.3% to 12.5%.

Likewise, the Dr Oz Good Life Adjustable Base Pro [27] provides an example of a mattress designed for PT, ensuring a head incline. Another class is that of bulky devices: a vest-type device (Bio Sleep Medical, Seoul, Republic of Korea) [21] and the Zzoma^®^ (Sleep Specialists, LLC, Abington, PA) bulky fin [29] were studied in this review. Their aim is to keep patients comfortably positioned on their side and prevent them from assuming the supine position. Vibration alarms, as the SPT [19] and the Buzz-POD^®^ (Gorman ProMed Pty Ltd., Victoria, Australia) supine alarm device [20] also share the goal of supine position avoidance, by providing a subtle vibration to ensure a timely response from the subject.

However, frequent arousals caused by supine alarm devices can lead to sleep fragmentation, resulting in subjective fatigue, reduced cognitive performance, and daytime sleepiness despite adequate total sleep time. PSG shows that this fragmentation correlates with disrupted sleep architecture, evidenced by reduced sleep efficiency and decreased time spent in REM and NREM sleep, particularly the N3 stage [30].

Deep NREM sleep is considered the most restorative sleep stage, crucial for sleep quality and maintenance. Disrupting slow-wave sleep experimentally has been shown to increase shallow sleep and sleep fragmentation, leading to greater daytime sleepiness and impaired daytime functioning [31]. A decrease in N3 sleep, in particular, is linked to poor performance on the Epworth Sleepiness Scale (ESS). Subjects deprived of N3 sleep frequently report snoring, excessive fatigue, daytime sleepiness, difficulty paying attention, and insomnia [32].

For these reasons it is essential to carry out adequate training with PT or changing position in order to improve adaptation and reduce sleep fragmentation. To decrease the impact of such a stress factor, the suggested solution should gradually be introduced. It could, for example, slowly train patients to avoid the supine position with a step by step buildup and a slow increase in vibration stimuli events. This slow increase in the amount of time spent in the non-supine positions should allow users to gently get used to their new way of sleeping, avoiding stress [30].

The pathophysiology behind the snoring improvement via head elevation is subtended by an increase in maximum cross-sectional area and a decreased closing pressure at both retropalatal and hypoglossal airways when compared to the neutral position. Moreover, experimentally obtained static pressure-area curves of the sniffing position exceed those of the natural position [33]. Plus, the height of devices imparting the head elevation is instrumental in promoting UA alignment and ensuring its patency with ease [24]. Moreover, head extension correlates with a “domino effect”, where the occipital condyles pushing forward ultimately result in lifting the mandible away from the throat [34]. It also facilitates the action of genioglossi muscles.

The supine position is associated with the longest and most burdensome snoring events, while snorers were found to have the shortest and fewest snoring events in the lateral decubitus position [35]. In particular, the right lateral decubitus appears to correlate with lesser, yet longer and louder snoring events compared to the left lateral decubitus [35].

In a 2014 article, Joosten et al. proposed four different causes leading to greater collapse of the UA in the supine position: force of gravity; decreased pulmonary volumes in the supine position; decreased tone of the genioglossus muscle during sleep; and critical pressure of pharyngeal closure (PCrit), which increases in the supine position compared to the lateral position.

Loud snoring is the primary clinical characteristic of OSA. It frequently lasts for a long time and is more difficult for the bed partner than it is for the patient. A very suggestive description of an OSA patient is four or five loud snores, followed by silence (apnea) and another series of loud snores. The onset of vomiting is often accompanied by severe, loud breathing (also referred to as “gasping”). These symptoms usually show up more when lying down or right after drinking alcohol. But occasionally, even in cases of severe sleep apnea, snoring might not be as noticeable. Additionally, there is mounting evidence that, even in the absence of OSA, snoring may contribute to daytime sleepiness. This could be explained by either UA inflammation brought on by vibrations in the pharynx caused by snoring or by UA resistance, which is characterized by episodes of increased respiratory effort followed by arousals and daytime sleepiness. The term non-apneic snorers is used to define snorers without any apneic events. The total snoring index is defined as the number of snore events in any body position (prone, supine, left, right, and upright) per hour of sleep. Non-apneic snorers were defined as patients with a snoring index > 1 and an AHI < 5. According to Benoist et al., 65.8% of non-apneic snorers were supine dependent [30,31].

Benoist et al. [31], found that the higher a patient’s BMI, the more likely they were to be position-dependent snorers. This result, however, is within a sample group of non-apneic patients. Other studies looking at the association of BMI with the position in apneic patients found that OSA seemed to correlate with a slightly reduced BMI [36]. In these studies, however, an increased BMI also correlated with an increased AHI. These findings, therefore, corroborate Mador et al.’s findings that patients with less OSA were more likely to be position dependent [37,38]. Nevertheless, the presence of snoring may herald an impending OSA since the two share common pathophysiological grounds [34,35].

However, both PT and HOBE may have some limitations. Some patients have trouble falling asleep on their sides. Reasons include chronic prone sleepers who use CPAP and patients with ankylosing spondylitis who have shoulder or hip complaints and/or back pain [39].

Furthermore, patients did not tolerate sleeping with an excessive elevation of the head and trunk for extended periods of time, so HOBE positions greater than 35° may have limited clinical applicability [12].

Both non-surgical treatments fall within the multimodal OSA therapy. It is crucial for sleep surgeons to know all the different possible options to treat snorers and OSA patients, always considering the possibility of integrating the different treatments with each other. Moreover, it is crucial that each treatment proposed by ENT in clinical practice must be supported by adequate scientific evidence and not a simple commercial product and must be followed-up in time. Finally, there are no studies comparing HOBE and PT to this day, and their lack marks a substantial unmet need in the challenging field of snoring management.

Reducing the intensity of snoring is imperative and goes beyond a mere aesthetic concern since snorers present an increased risk of developing sudden sensorineural hearing loss, affecting predominantly the high frequencies, which reflects primarily in the inability to distinguish speech in noisy environments [40,41]. Repeated loud snoring sound generated by the vibration of the overlying palate is transmitted through the eustachian tube, thereby reaching and damaging the cochlea; hence, the impairment in high-frequency hearing [42,43].

While some may argue that hearing loss is solely caused by concomitant hypoxemic damage to the acoustic nerve [44], bed partners appear to display a unilateral high-frequency pattern of hearing loss consistent with noise-induced hearing loss, affecting the ear chronically exposed to snoring noise [45]. This is probably suggestive of a complex interplay of factors, which eventually results in hearing impairment [41,46]. In clinical practice, snoring is hardly ever abolished. Therefore, even a reduction in snoring intensity represents an achievement per se in the complex scenario of snoring management.

## 5. Conclusions

The findings presented in this review showed the effect of changing position on snoring. PT and HOBE can be considered as reliable therapeutic options for improving snoring in snorers and OSA patients. However, there are only very few short-term recent studies on the effectiveness of these devices. In order to confirm and improve these encouraging results, there is an urgent need for dedicated controlled trials to test the clinical benefits of changing position on snoring in a large patient population.

## Figures and Tables

**Table 1:** Demographic characteristics of the patients included in the studies.

Devices for Positional Therapy									
Author (Year)	Country	Study Design	N. Patients	Age	Sex (M/F)	Snoring/OSA	BMI (kg/m^2^)	Neck Circumference (cm)	**Follow-Up**
Benoist 2018 [19]	Netherlands	Non-controlled study	30	41.5 (34.0–51.3)	15/15	30 snorers	25.0 (22.5–28.3)	-	6 weeks
Bignold 2011 [20]	Australia	interventional, controlled, and randomized crossover study.	15	58.2 ± 13.9	13/2	15 positional OSA patients with snoring	28.8 ± 2.5	-	3 weeks
Choi 2009 [21]	Korea	Comparative parallel study	17	50.1 ± 17.6	13/4	17 snorers with or without mild OSA	-	-	-
**Active pillows**									
**Author (year)**	**Country**	**Study design**	**N. patients**	**Age**	**Sex (M/F)**	**Snoring/OSA**	**BMI (kg/m^2^)**	**Neck circumference (cm)**	**Follow-up**
Cazan 2017 [22]	Germany	interventional, controlled, and randomized crossover study.	20	41.7 ± 7.8	16/4	20 snorers	<30	-	4 weeks
Chung 2021 [23]	China	single-center, single-treatment, non-controlled, non-randomized study	30	59.30 ± 12.93	15/15	23 mild to moderate OSA	-	-	-
Chen 2015 [24]	China	Prospective study	25	47.0 (32.0–53.0)	21/4	-	24.8 (23.1–26.4)	38.0 (36.0–40.0)	-
Newell 2018 [25]	Belgium	Prospective study	28	51.5 ± 10.8	17/11	-	28.9 ± 4.6	39.6 ± 3.0	6 months
**Head and body inclination**									
**Author (year)**	**Country**	**Study design**	**N. patients**	**Age**	**Sex (M/F)**	**Snoring/OSA**	**BMI (kg/m^2^)**	**Neck circumference (cm)**	**Follow-up**
Iannella 2022 [12]	Italy	Prospective study	45	46.4 ± 14.5	39/6	-	27.2 ± 3.1	-	-
Lee 2024 [26]	Korea	Randomized, single-blind, parallel two-arm comparative study	32	Study group: 34.8 ± 5.4 Control group: 34.8 ± 5.5	Study group: 10/2 Control group: 9/2	-	Study group: 26.0 ± 4.0 Control group: 25.6 ± 3.6	-	2 weeks
Danoff-Burg 2022 [27]	USA	Prospective study	25	38 ± 11.38 (21–62)	15/10	25	<30	-	8 weeks

**Table 2:** Outcomes of the included studies.

Devices for Positional Therapy												
Author (Year)	Treatment Device	% of Time Sleeping Supine	AHI			ODI	RDI	Mean SO_2_	Snoring			Questionnaires
			Total	Supine	Non-Supine				Total	Supine	**Non-Supine**	
Benoist 2018 [19]	Sleep position trainer (SPT)	baseline: 40.3 (24.7–50.4)	baseline: 2.5 (1.2–3.4)	-	-	-	-	-	-	baseline: 414.8 (252.8–699.5) events/h	baseline: 205.9 (115.7–503.9) events/h	Snore Outcome Survey (SOS): baseline: 35.0 ± 13.5 post: 54.4 ± 17.8 Spouse/Bed Partner Survey (SBPS): baseline: 26.0 ± 16.2 post: 52.7 ± 25.1 VAS baseline: 7.6 ± 1.4 post: 6.2 ± 2.4
Bignold 2011 [20]	Alarm device (Buzz- POD, Gorman ProMed Pty Ltd., Victoria, Australia)	baseline: 36.4 ± 20.6	24.1 ± 10.5	51.3 ± 23.3	9.7 ± 3.9	baseline: 5.5; follow-up: 3.4	-	-	active treatment: 140 events/h; inactive treatment: 180 events/h	-	-	-
Choi 2009 [21]	Vest with inflatable chambers	baseline: 67.1 post: 25.0	Baseline: 7.7 ± 7.0 Post: 4.8 ± 8.1	-	-	Baseline: 8.0 ± 7.4 Post: 6.2 ± 11.2	-	baseline: 96.3 ± 1.0 post: 95.8 ± 1.4	baseline: 36.7 ± 20.6% post: 15.7 ± 16.2%	baseline: 45.8 ± 22.8% post: 25.4 ± 20.6%	baseline: 16.8 ± 23.7% post: 12.0 ± 14.0%	refreshment once awake: 5.9 ± 2.1 no pains in the back or waist: 6.2 ± 2.4 reduction in sleep disordered breathing: 5.9 ± 2.2
**Active pillows**												
**Author (year)**	**Treatment device**	**% of time sleeping supine**	**AHI**			**ODI**	**RDI**	**Mean SO_2_**	**Snoring**			**Questionnaire**
			**Total**	**Supine**	**Non-Supine**				**Total**	**Supine**	**Non-supine**	
Cazan 2017 [22]	The pillow Sissel^®^ (Sissel Novacare, Bad Dürkheim, Germany)	-	baseline: 7.34 ± 7.43 post: 6.91 ± 6.71	baseline: 9.14 ± 15.23 post: 8.62 ± 12.77	-	-	baseline: 19.27 ± 16.68 post: 20.39 ± 16.47	-	baseline: 269 ± 249.7 events/h post: 162.5 ± 169.8 events/h	-	-	VAS baseline: 7.2 post: 4.0
Chung 2021 [23]	smart anti-snore pillow (SAP)	baseline: 74.3 ± 23.7 SAP: 73.1 ± 24.8	baseline: 21.8 ± 15.7 SAP: 16.5 ± 17.8	baseline: 27.3 ± 17.5 SAP: 20.4 ± 19.9	baseline: 4.0 ± 6.3 SAP: 4.1 ± 9.3	baseline: 15.8 ± 16.3 SAP: 7.8 ± 2.5	-	baseline: 91.0 ± 17.3; SAP: 94.2 ± 2.3	baseline: 501.5 ± 235.1 events/h SAP: 360.9 ± 218.1 events/h	-	-	-
Chen 2015 [24]	head-positioning pillow (HPP); Power Sleep^®^ anti-snore pillow	-	baseline: 7.0 (6.0, 15.2)	baseline: 10.1 (6.9, 22.0)	baseline: 2.2 (0.9, 3.7)	baseline: 4.2 (1.5, 8.9) HPP: 3.5 (1.6, 8.5)	-	baseline: 95.8 (95.1, 96.1) HPP: 96.1 (95.2, 96.8)	Snoring index: baseline: 218.0 (100.0, 288.5) events/h HPP:115.0 (48.0, 260.3) events/h Snoring intensity: baseline: 74.2 (68.4, 81.1) dB HPP: 74.3 (66.7, 80.4) dB	-	-	-
Newell 2018 [25]	Sleep-positioning pillow (Posiform^®^)	baseline: 47.5 ± 21.2 1 night:19.2 (10.6–41.8) 1 month: 358.4 ± 115.1	baseline: 12.1 ± 3.8 1 night: 6.4 (3.9; 9.8) 1 month: 6.0 (3.5; 13.0)	baseline:25.2 ± 13.7 1 night: 14.6 (5.9; 24.6) 1 month: 23.0 ± 18.4	baseline: 3.4 ± 2.7 1 night: 3.3 ± 2.0 1 month: 3.7 ± 3.4	baseline: 6.1 ± 3.1 1 night: 2.8 (1.8; 4) 1 month: 3.4 (1.4; 6.6)	baseline: 18.4 ± 5.6 1 night: 11.5 ± 4.6 1 month: 12.9 ± 8.4	baseline: 93.8 ± 1.6 1 night: 94.8 ± 1.5 1 month: 94.0 (94.0; 95.0)	baseline: 26.3 ± 18.6% 1 night: 21.5 ± 17.1% 1 month: 20.4 ± 22.6%	baseline: 40.8 ± 25.1% 1 night: 35.0 ± 28.1% 1 month: 28.3 ± 27.7%	baseline: 9.5 ± 12.2% 1 night: 15.2 ± 16.3% 1 month: 14.1 ± 20.6%	Epworth Sleepiness Scale (ESS) baseline: 11.1 ± 5.1; 1 month: 8.0 ± 4.6, Fatigue severity scale (FSS): baseline: 4.0 ± 1.3 1 month: 3.5 ± 1.7, Pittsburgh Sleep Quality Index (PSQI) baseline: 7.4 ± 3.2 1 month: 4.6 ± 21.1 Function Outcomes of Sleep Questionnaire (FOSQ) baseline: 14.8 ± 3.2 1 month: 17.1 ± 2.4
**Head and body inclination**												
**Author (year)**	**Treatment device**	**% of time sleeping supine**	**AHI**			**ODI**	**RDI**	**Mean SO_2_**	**Snoring**			**Questionnaire**
			**Total**	**Supine**	**Non-supine**				**Total**	**Supine**	**Non-supine**	
Iannella 2022 [12]	Head-of-bed-elevation positioning (HOBE)	-	baseline: 26.2 ± 9.9 HOBE: 17.7 ± 12.4	-	-	baseline: 21.2 ± 10 HOBE: 16.1 ± 11.7	-	baseline: 92 ± 3.3 HOBE: 93.7 ± 2.2	baseline: 17.3 ± 11.5 HOBE: 12.5 ± 12.6	-	-	Did you sleep well last night? YES (37) Have you noticed any differences between the second part of sleep in an elevated position and the first part in supine position? YES (10) Do you think that can you can sleep every night with a HOBE? YES (34)
Lee 2024 [26]	Mild head of bed elevation of 7.5 degree		Baseline: Study: 13.6 [2.2–32.1] Control: 2.9 [1.7–22.9] Post: Study: 10.8 ± 2.3 Control: 18.5 ± 2.4	-	-	-	Baseline: Study: 21.4 [2.8–32.1] Control: 8.6 [3.5–29.7] Post: Study: 14.0 ± 2.2 Control: 21.7 ± 2.3	Baseline: Study: 95.3 [93.2–96.0] Control: 96.0 [95.1–96.4] Post: Study: 95.6 ± 0.2 Control: 95.1 ± 0.2	Baseline: Study: 17.8% [3.6–38.2] Control: 8.8% [1.8–23.7] Post: Study: 1.92 ± 3.1% Control: 24.4 ± 3.2%	-	-	-
Danoff-Burg 2022 [27]	Adjustable bed base, upper body at a 12-degree incline	-	-	-	-	-	-	-	baseline: 9.28 ± 4.29% post: 8.61 ± 3.64	-	-	Perceived time snoring: baseline: 6 nights per week post: 5 nights per week Time woken up: baseline: sometimes post: rarely Partner woke them less often to stop snoring: baseline: sometimes post: rarely

## Data Availability

The original contributions presented in the study are included in the article, further inquiries can be directed to the corresponding author.

## References

[1] Olszewska E., De Vito A., Baptista P., Heiser C., O’connor-Reina C., Kotecha B., Vanderveken O., Vicini C. (2024). Consensus Statements among European Sleep Surgery Experts on Snoring and Obstructive Sleep Apnea: Part 1 Definitions and Diagnosis. J. Clin. Med..

[2] Ieto V., Kayamori F., Montes M.I., Hirata R.P., Gregório M.G., Alencar A.M., Drager L.F., Genta P.R., Lorenzi-Filho G. (2015). Effects of Oropharyngeal Exercises on Snoring: A Randomized Trial. Chest.

[3] Moffa A., Giorgi L., Carnuccio L., Cassano M., Lugo R., Baptista P., Casale M. (2023). Barbed Pharyngoplasty for Snoring: Does It Meet the Expectations? A Systematic Review. Healthcare.

[4] Chan C.H., Wong B.M., Tang J.L., Ng D.K. (2012). Gender difference in snoring and how it changes with age: Systematic review and meta-regression. Sleep Breath..

[5] Chuang L.-P., Lin S.-W., Lee L.-A., Li H.-Y., Chang C.-H., Kao K.-C., Li L.-F., Huang C.-C., Yang C.-T., Chen N.-H. (2017). The gender difference of snore distribution and increased tendency to snore in women with menopausal syndrome: A general population study. Sleep Breath..

[6] Sleep Disorders, An Issue of Neurologic Clinics, Volume 30-4—1st Edition|Elsevier Shop. https://shop.elsevier.com/books/sleep-disorders-an-issue-of-neurologic-clinics/vaughn/978-1-4557-4951-5.

[7] Blumen M., Salva M.A.Q., D’Ortho M.-P., Leroux K., Audibert P., Fermanian C., Chabolle F., Lofaso F. (2009). Effect of sleeping alone on sleep quality in female bed partners of snorers. Eur. Respir. J..

[8] Yaremchuk K. (2020). Why and When to Treat Snoring. Otolaryngol. Clin. N. Am..

[9] Lechat B., Naik G., Appleton S., Manners J., Scott H., Nguyen D.P., Escourrou P., Adams R., Catcheside P., Eckert D.J. (2024). Regular snoring is associated with uncontrolled hypertension. NPJ Digit. Med..

[10] Sowho M., Sgambati F., Guzman M., Schneider H., Schwartz A. (2019). Snoring: A source of noise pollution and sleep apnea predictor. Sleep.

[11] Moffa A., Rinaldi V., Mantovani M., Pierri M., Fiore V., Costantino A., Pignataro L., Baptista P., Cassano M., Casale M. (2020). Different barbed pharyngoplasty techniques for retropalatal collapse in obstructive sleep apnea patients: A systematic review. Sleep Breath..

[12] Iannella G., Cammaroto G., Meccariello G., Cannavicci A., Gobbi R., Lechien J.R., Calvo-Henríquez C., Bahgat A., Di Prinzio G., Cerritelli L. (2022). Head-of-Bed Elevation (HOBE) for Improving Positional Obstructive Sleep Apnea (POSA): An Experimental Study. J. Clin. Med..

[13] Bin Hsu Y., Lan M.Y., Huang Y.C., Huang T.T., Lan M.C. (2021). Effect of back-up head-elevated position during drug-induced sleep endoscopy in obstructive sleep apnea patients. Sleep Breath..

[14] Kaltenbach T., Crockett S., Gerson L.B. (2006). Are lifestyle measures effective in patients with gastroesophageal reflux disease? An evidence-based approach. Arch. Intern. Med..

[15] Llaurado-Serra M., Ulldemolins M., Güell-Baró R., Coloma-Gómez B., Alabart-Lorenzo X., López-Gil A., Bodí M., Rodriguez A., Jiménez-Herrera M. (2015). Evaluation of head-of-bed elevation compliance in critically ill patients under mechanical ventilation in a polyvalent intensive care unit. Med. Intensive.

[16] Spooner A.J., Corley A., Sharpe N.A., Barnett A.G., Caruana L.R., Hammond N.E., Fraser J.F. (2014). Head-of-bed elevation improves end-expiratory lung volumes in mechanically ventilated subjects: A prospective observational study. Respir. Care.

[17] Moher D., Liberati A., Tetzlaff J., Altman D.G. (2009). Preferred reporting items for systematic reviews and meta-analyses: The PRISMA statement. PLoS Med..

[18] O’Sullivan D., Wilk S., Michalowski W., Farion K. (2013). Using PICO to Align Medical Evidence with MDs Decision Making Models. Stud. Health Technol. Inform..

[19] Benoist L.B.L., Beelen A.M.E.H., Torensma B., de Vries N. (2018). Subjective effects of the sleep position trainer on snoring outcomes in position-dependent non-apneic snorers. Eur. Arch. Oto-Rhino-Laryngol..

[20] Bignold J.J., Mercer J.D., Antic N.A., McEvoy R.D., Catcheside P.G. (2011). Accurate Position Monitoring and Improved Supine-Dependent Obstructive Sleep Apnea with a New Position Recording and Supine Avoidance Device. J. Clin. Sleep Med..

[21] Choi J.H., Park Y.H., Hong J.H., Kim S.J., Park D.S., Miyazaki S., Lee S.H., Shin C., LEE J. (2009). Efficacy study of a vest-type device for positional therapy in position dependent snorers. Sleep Biol. Rhythm..

[22] Cazan D., Mehrmann U., Wenzel A., Maurer J.T. (2017). The effect on snoring of using a pillow to change the head position. Sleep Breath..

[23] Te Chung T., Lee M.T., Ku M.C., Yang K.C., Wei C.Y. (2021). Efficacy of a Smart Antisnore Pillow in Patients with Obstructive Sleep Apnea Syndrome. Behav. Neurol..

[24] Chen W.-C., Lee L.-A., Chen N.-H., Fang T.-J., Huang C.-G., Cheng W.-N., Li H.-Y. (2015). Treatment of snoring with positional therapy in patients with positional obstructive sleep apnea syndrome. Sci. Rep..

[25] Newell J., Mairesse O., Neu D. (2018). Can positional therapy be simple, effective and well tolerated all together? A prospective study on treatment response and compliance in positional sleep apnea with a positioning pillow. Sleep Breath..

[26] Lee S., Park J.H., Kim J.Y., Park S.-W., Shin H.-B., Kim B.H. (2024). Implementation of Head of Bed Elevation Using Adjustable Bed and Its Effects on Sleep: A Pilot Randomized Trial. Altern. Ther. Health Med..

[27] Danoff-Burg S., Rus H.M., Weaver M.A., Raymann R.J.E.M. (2022). Sleeping in an Inclined Position to Reduce Snoring and Improve Sleep: In-home Product Intervention Study. JMIR Form. Res..

[28] Adnet F., Baillard C., Borron S.W., Denantes C., Lefebvre L., Galinski M., Martinez C., Cupa M., Lapostolle F. (2001). Randomized study comparing the ‘sniffing position’ with simple head extension for laryngoscopic view in elective surgery patients. Anesthesiology.

[29] Permut I., Diaz-Abad M., Chatila W., Crocetti J., Gaughan J.P., D’Alonzo G.E., Krachman S.L. (2010). Comparison of Positional Therapy to CPAP in Patients with Positional Obstructive Sleep Apnea. J. Clin. Sleep Med..

[30] Benkirane O., Delwiche B., Mairesse O., Peigneux P. (2022). Impact of Sleep Fragmentation on Cognition and Fatigue. Int. J. Environ. Res. Public Health.

[31] Dijk D.-J. (2009). Regulation and Functional Correlates of Slow Wave Sleep. J. Clin. Sleep Med..

[32] Basunia M., Fahmy S.A., Schmidt F., Agu C., Bhattarai B., Oke V., Enriquez D., Quist J. (2016). Relationship of symptoms with sleep-stage abnormalities in obstructive sleep apnea-hypopnea syndrome. J. Community Hosp. Intern. Med. Perspect..

[33] Isono S., Tanaka A., Ishikawa T., Tagaito Y., Nishino T. (2005). Sniffing Position Improves Pharyngeal Airway Patency in Anesthetized Patients with Obstructive Sleep Apnea. Anesthesiology.

[34] Makofsky H.W. (1997). Snoring and obstructive sleep apnea: Does head posture play a role?. Cranio—J. Craniomandib. Sleep Pract..

[35] Huang Z., Lobbezoo F., Vanhommerig J.W., Volgenant C.M., De Vries N., Aarab G., Hilgevoord A.A. (2023). Effects of demographic and sleep-related factors on snoring sound parameters. Sleep Med..

[36] Oksenberg A., Silverberg D.S., Arons E., Radwan H. (1997). Positional vs nonpositional obstructive sleep apnea patients: Anthropomorphic, nocturnal polysomnographic, and multiple sleep latency test data. Chest.

[37] Mador M.J., Kufel T.J., Magalang U.J., Rajesh S.K., Watwe V., Grant B.J.B. (2005). Prevalence of positional sleep apnea in patients undergoing polysomnography. Chest.

[38] Itasaka Y., Miyazaki S., Ishikawa K., Togawa K. (2000). The influence of sleep position and obesity on sleep apnea. Psychiatry Clin. Neurosci..

[39] Ravesloot M.J.L. (2024). Positional Treatment of Obstructive Sleep Apnea. Otolaryngol. Clin. N. Am..

[40] Simsek A., Aslan M. (2024). Evaluation of the auditory findings of patients with obstructive sleep apnea syndrome. Am. J. Otolaryngol..

[41] Manuele C., Antonio M. (2023). Ear, Nose, and Throat (ENT) Aspects of Obstructive Sleep Apnea (OSA). Obstructive Sleep Apnea.

[42] Kayabasi S., Hizli O., Yildirim G. (2019). The association between obstructive sleep apnea and hearing loss: A cross-sectional analysis. Eur. Arch. Oto-Rhino-Laryngol..

[43] Lee J.M., Lee H.J. (2023). Is sleep apnea truly associated with hearing loss? A nationwide, population-based study with STOP-BANG questionnaire. Front. Public Health.

[44] Lim C.C., Ahmad T.E.B.T.N., Bin Sawali H., Bin Afandi A.N., Paniselvam V., Bernard M.W., Narayanan P., Bin Abu Bakar M.Z. (2023). Evaluation of auditory system in obstructive sleep apnea patients. Eur. Arch. Oto-Rhino-Laryngol..

[45] Sardesai M.G., Tan A.K.W., Fitzpatrick M. (2003). Noise-induced hearing loss in snorers and their bed partners. J. Otolaryngol..

[46] Ekin S., Turan M., Arısoy A., Gunbatar H., Sunnetcioglu A., Asker S., Yıldız H. (2016). Is There a Relationship Between Obstructive Sleep Apnea (OSA) and Hearing Loss?. Med. Sci. Monit..

